# Bacterial mechanosensitive channels: progress towards an understanding of their roles in cell physiology^☆^

**DOI:** 10.1016/j.mib.2014.01.005

**Published:** 2014-04

**Authors:** Ian R Booth

**Affiliations:** 1 School of Medical Sciences, University of Aberdeen, Aberdeen, AB25 2ZD, United Kingdom; 2 Visiting Associate in Chemistry, California Institute of Technology, Pasadena, CA 91125, United States

## Abstract

Bacterial mechanosensitive channels sense the changes in lateral tension in the bilayer of the cytoplasmic membrane generated by rapid water flow into the cell. Two major structural families are found widely distributed across bacteria and archaea: MscL and MscS. Our understanding of the mechanisms of gating has advanced rapidly through genetic analysis, structural biology and electrophysiology. It is only recently that the analysis of the physiological roles of the channels has kept pace with mechanistic studies. Recent advances have increased our understanding of the role of the channels in preventing structural perturbation during osmotic transitions and its relationship to water flow across the membrane. It is to these recent developments that this review is dedicated.

## Highlights

• Multiple mechanosensitive channels are found in most bacteria and archaea.
• Channels are required to prevent loss of structural integrity during transitions from high to low osmolarity.
• Channel diversity feeds into the detailed response of cells to hypo-osmotic stress.
• There is growing evidence that organisms have evolved MS channels that reflect their niche.
• Structural diversity may reflect roles additional to the observed function of protection of structural integrity.

**Current Opinion in Microbiology** 2014, **18**:16–22

This review comes from a themed issue on **Cell regulation**

Edited by **Cecília Maria Arraiano** and **Gregory M Cook**

For a complete overview see the Issue and the Editorial

Available online 6th March 2014

1369-5274/$ – see front matter, © 2014 The Authors. Published by Elsevier Ltd. All rights reserved.

**http://dx.doi.org/10.1016/j.mib.2014.01.005**

## Introduction

Mechanosensitive (MS) channels sense changes in the tension in the lipid bilayer of the cytoplasmic membrane [1^•^]. Bacterial channels have been well-studied in a range of organisms [2^•^] and they are considered to be useful models for mechanotransduction in higher organisms [3]. Mammalian channels are frequently ion-selective and thus generate specific signals that are integrated by the neuronal system leading to an altered behaviour. In contrast, bacterial mechanosensitive channels are generally non-specific in terms of the ions and molecules that pass through the open pore. Their transition from the closed to the open state creates a transient pore of quite large dimensions, minimally ≥6 Å diameter (the size of a hydrated K^+^ ion) through to ∼30 Å diameter for MscL [2^•^]. Their proposed major role in cell physiology is well-established, namely protection of the physical integrity of the cell during transitions from high osmolarity to low [4]. One of the most important questions remaining addresses channel abundance, structural diversity and plurality in bacterial species. This short article will review the timing of channel gating and its importance for the roles of the channels.

## Osmoregulation and cytoplasmic solute concentrations

**Figure 1:**
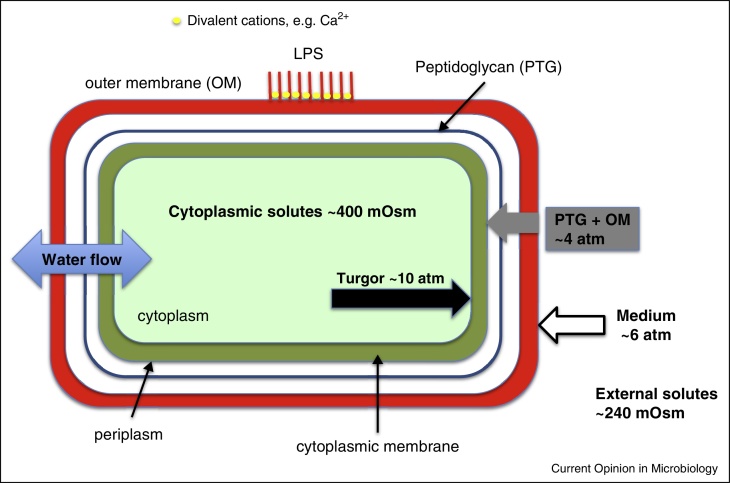
The generation of turgor and resistance to the force. In *E. coli* cells growing in a medium of ∼240 mOsm (a standard minimal medium or LB containing 5 g/L NaCl) one may confidently expect to find ∼200 mm cytoplasmic anions and ∼300 mm K^+^. Approximately 100 mm of the K^+^ matches fixed anions and is thus not considered for the calculation of the outward turgor of ∼10 atm. Given the medium contributes ∼6 atm the net turgor pressure is ∼4 atm. MS channels will gate if there is a net outward pressure of ∼0.1 atm and thus the cell wall and outer membrane, between them, contribute a resistance of ≥4 atm to maintain MS channels closed. There are at least two contributions to the strength of the cell wall — the first, already described, is the crosslinking of the peptidoglycan and the second is the outer membrane that can provide some resistive force through the binding together of the lipopolysaccharide chains by divalent cations [46].

Bacterial cells accumulate solutes in their cytoplasm well beyond the concentrations that might be required for metabolism. In most bacteria there is a preference for the accumulation of potassium and glutamate [5]. However, diverse metabolic anions accumulate to millimolar levels, such that the cytoplasm may contain as much as 200 mM osmotically active anions even when grown at moderately low osmolarity (∼240 mOsm) [6]. This would generate a net turgor pressure of ∼4 atm (∼40 mOsm solute ∼ 1 atm [2^•^]; the osmolarity of the medium is equivalent to ∼6 atm) directed outwards from the cell (Figure 1). Movement of water across the membrane into the cytoplasm generates the turgor pressure and provides the expansion space required for growth through biosynthesis of new polymers. Measurement of turgor pressure is extremely difficult and there is no certainty that this parameter does not vary with either growth conditions or with the identity of the organism. A net outward pressure of 4 atm in *E. coli* cells was suggested [7], but recent experiments have questioned this [8^•^].

When subjected to hyperosmotic stress, Gram negative bacteria exhibit a biphasic strategy to counter water loss. In the initial phase potassium and glutamate pools increase and subsequently these ions may be replaced by compatible solutes, such as trehalose, betaine and proline [5]. A cell adapted to high osmolarity is at risk when transferred to low osmolarity due to the osmotically driven water flow into the cytoplasm. A decrease in the external osmolarity of 800 mOsm (equivalent of transfer from growth medium containing 0.5 m NaCl into growth medium alone or approximating the transfer of cells from sea water to fresh water) could raise the turgor pressure by 20 atm [2^•^]. The actual increase experienced by the cells depends on the rate of water penetration into the cytoplasm, the elasticity of the peptidoglycan (PTG) and the activity of mechanosensitive channels.

## The centrality of water in life

Central to understanding the core physiology of bacterial MS channels is an appreciation of the rapidity of water fluxes across the lipid bilayer. The membrane bilayer is highly permeable to water and in some bacterial species this natural permeability is further augmented by expression of aquaporins [9]. In response to hyperosmotic shock [10,11] (J Mika, PhD Thesis, Groningen, 2012) and hypoosmotic shock [12^••^] the cell shrinks or expands, respectively, on the very rapid timescales (30% volume change in <1 s is typical). A bacterial cell of 10^−15^ L contains ∼3–4 × 10^10^ water molecules. Considering an *E. coli* cell as a cylinder (∼2 μm length and 1 μm diameter) that can expand along its length but not readily change its diameter (at least over the very short timescales associated with osmotically driven water movements), which is consistent with current theories of peptidoglycan structure [13^•^], an expansion of ∼12% [14] would require ∼4–5 × 10^9^ water molecules to cross the membrane, which, in *E. coli*, can occur in 100 ms [10,11] (J Mika, PhD Thesis, Groningen, 2012). The capacity to withstand rapid water movements is dependent upon the operation of MS channels and on the strength of the cell wall.

Peptidoglycan, which gives the cell its physical integrity and shape [13^•^], is a dynamic, semi-elastic polymer constructed from oligosaccharides of varying lengths (N-acetylglucosamine and N-acetylmuramic acid pentapeptide units; NAG-NAM-p5) crosslinked by short peptides. Sugar chains are organised principally in the circumferential direction, while the peptides are oriented in the long direction of the cell [13^•^,15,16,17]. Peptidoglycan is not a continuous structure; the sugar chains are of variable length (a single circumference requiring many independent polysaccharide chains) and the peptide crosslinking is incomplete [18]. This variation creates a mesh in which there are holes (of varying sizes) that are bounded by the sugars and peptides [18]. Growth of *E. coli* cells is largely by extension in the long direction and this requires the peptidoglycan be a highly dynamic structure; increasing the length of the cell is principally achieved by breakage of the peptide bonds and the insertion of new wall material [13^•^]. Although the peptidoglycan is a unique structure between the cytoplasmic and the outer membranes, there are important connections to both membranes through synthetic complexes and lipoproteins, respectively [13^•^]. Some of these connections are transient, but others, for example, lipoprotein linkages are covalent bonds to the PTG peptides. The dynamic nature of the peptidoglycan renders the cell susceptible to physical disruption by rapid water flow into the cytoplasm.

## Expansion of the cell, some considerations

The effect of increased water influx into the cytoplasm is conditional on the pre-existing state of the peptidoglycan. Measurements have been made, by atomic force microscopy, of the expansion that isolated PTG sacculi can undergo in response to applied force [14]; a ∼12% expansion was measured for every 1 atm of pressure applied. If the cell can expand under the inflow of water there will be no net increase in pressure on the membrane provided that the bilayer can increase its surface area on the same timescale as the water movements. However, the membrane has a limited expansive capacity due to a lack of extensive phospholipid reserves — estimates suggest 2–4% expansion, by increasing the distance between headgroups of the phospholipids, as a mechanical upper limit [19]. Moreover, rapidly growing cells are likely to have their PTG sacculus already stretched and it is not clear whether an immediate expansion on this scale is feasible without imposing considerable strain on the wall.

**Figure 2:**
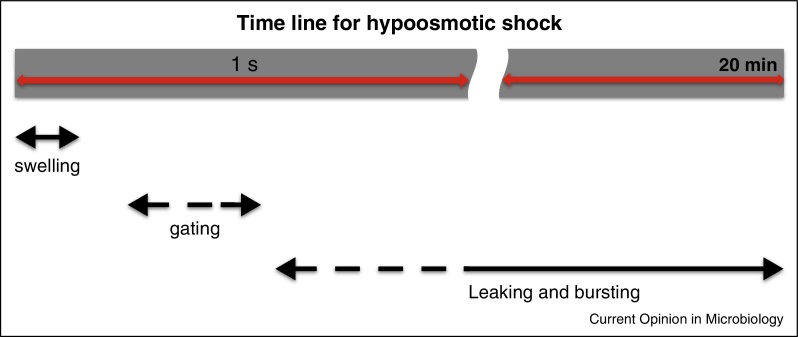
Timeline for swelling, adaptation or death. This figure illustrates that, from measurements by stopped flow and microscopy, swelling was observed ∼30–50 ms after imposition of a severe (∼900 mOsm) hypoosmotic shock [12^••^]. Changes in refractive index consistent with channel gating suggested that this occurs between 150 and 200 ms after shock [12^••^]. Initial cell disruption events were first observed 200–1000 ms after downshock [33^••^] but lytic events continue for at least 20 min [4] (SS Black *et al.*, unpublished data; M Bialecka-Fornal *et al*., personal communication).

MS channels, when assayed in membrane patches, gate at an imposed pressure on the lipid bilayer (i.e. shorn of the cell wall) of around 0.1 atm (depending on the specific lipid context and the shape of the membrane patch) [20–22]. Clearly, therefore, as the PTG achieves its maximum expanded stable state a further increase in turgor pressure will lead to gating of the MS channel complement. Sukharev and colleagues [12^••^] measured the rapidity of the initial swelling upon imposition of a hypoosmotic shock, using the change in the refractive index of cells. They observed swelling to occur ∼30–50 ms after lowering the external osmolarity, followed by channel gating after ∼150–200 ms. Previous studies to detect the responsiveness of MscS and MscL to tension changes suggested that the channels gate ∼3–5 μs after the tension reaches the activation threshold [23]. This suggests that channel gating ∼100 ms after swelling is not due to intrinsic lack of responsiveness of the channels, but may be due to generation of the gating signal from a combination of water inflow, cell expansion and other, as yet unknown, modulations of the cell envelope. An approximate timeline for swelling, adaptation and death can be constructed (Figure 2) that allows one to appreciate the extreme rapidity of the onset of hypoosmotic stress and the speed of the response required.

## Cell death follows multiple paths

Failure of mechanosensitive channels to open leads to cell death [4,24,25,26^•^]. The precise fate of individual cells lacking MS channels is complex. Cell fate is determined by a combination of known and unknown parameters that vary between individuals. For each cell these include the turgor pressure, the number of channels and the strength of the cell wall. In addition, the rate of change of the osmolarity is itself a major determinant of cell fate [27^••^] (see below). Colony counts have been the preferred method to investigate the fate of channel-less mutants and such studies have been informative in defining the core role of mechanosensitive channels [4,24,25,26^•^,28^••^]. These assays reveal that a small, but significant, fraction of mutant cells survive hypoosmotic shock. When such survivors are re-cultured they recapitulate the original pattern of survival, that is the majority die, but a few survive. Thus, the majority of survivors have not acquired protective mutations, but rather their lysis is prevented by some variation in the parameters described above. There is a reliable *qualitative* correlation between channel numbers, intrinsic channel properties (gating tension, open dwell time and conductance [29]) and survival of a sudden hypoosmotic shock [4,28^••^,29–31], which has provided a guide to researchers on the activity of their chosen channel [2^•^,27^••^,32].

Two recent developments have begun to prise open the pathways that cells follow during cell death. Simple conclusions about cell death have been drawn, using optical tweezers to study the fate of individual cells, in parallel with electron microscopy and FACS analysis of populations. Thus, the majority of *E. coli* cells lacking MscS, MscL and MscK (the three major mechanosensitive channels identified by patch clamping) ‘burst’ on sudden extreme hypoosmotic shock (∼900–1000 mOsm) and form cell-shaped ghosts that retain some nucleic acid but little protein [33^••^]. Individual cells behave in a number of experimentally distinguishable ways: some retain their integrity and remain phase dark (or fluorescent in the case of GFP-labelled cells), still others form transient lesions that allow the escape of small proteins (e.g. GFP) but retain their phase dark status indicating that they have retained the majority of proteins, and a third class burst leading to evacuation of the majority of cell proteins and form ghosts [33^••^]. Thus ms timescale events can be observed by analysis of single cells, but it is clear from population-based assays that lysis (assayed by protein release) continues for 10–20 min post-shock [4] (SS Black *et al.*, unpublished data).

Another recent study has investigated the fate of single cells in populations that are subjected to hypoosmotic shock at different rates (M Bialecka-Fornal *et al.*, personal communication). This experimental system has the benefit of controlled hypoosmotic shock imposed at a defined rate combined with the observation of large numbers of cells. Using *E. coli* mutants with different channel complements, it was observed that the rate of cell death depended on the identity of the channels retained in the mutant strains. This study clearly identifies a role for the ‘minor’ mechanosensitive channels and observed that the most severe survival defect was with a mutant that lacked all seven MS channel homologues, MJF641 [28^••^,34]. Again multiple pathways to cell death were observed with some cells simply failing to grow while others formed distinct membrane blebs prior to loss of phase dark character but with retention of overall shape (ghosts) (M Bialecka-Fornal *et al.*, personal communication).

These two studies frame our current understanding of the fate of cells lacking MS channels and make the case for their role in retention of structural integrity of the cell. Finally, there are nice parallels with antibiotic-induced killing via inhibition of PTG crosslinking, for example, using sublethal vancomycin [8^•^], that is coupled to growth. Here blebs of inner membrane penetrate through the PTG and outer membrane, but the rate of formation is essentially stochastic (M Bialecka-Fornal *et al.*, unpublished data) as is the case for blebs that form after hypoosmotic shock in MS channel-free mutants. Thus, the delayed release of protein [4] (SS Black *et al.*, unpublished data) and the formation of blebs (M Bialecka-Fornal *et al.*, personal communication) argue for damage that has occurred during the shock becoming manifest only when the cell starts to grow. Restoration of growth requires the recovery of solutes [35] to restore cell turgor that may drive expansion of the cytoplasmic membrane through the damaged wall.

## Channel diversity and adaptation to niche

It is well-established that there are two major classes of bacterial mechanosensitive channels, namely MscS and MscL, which were originally defined by electrophysiology in *E. coli* [2^•^,21] (Figure 1), but subsequently by their different structures [1^•^]. Subsequent analyses revealed six MscS homologues in *E. coli*, each of which has mechanosensitive channel activity, but the majority of these channels are not observed regularly in electrophysiological assays and play only minor, but still significant roles in protecting cells [4,28^••^,31]. The loss of the YbdG channel lowers the magnitude of the salt concentration at which death occurs after a sudden downshock [31] and the increases the rate dependence of cell death in controlled hypoosmotic shock (M Bialecka-Fornal *et al.*, personal communication). Genome sequences revealed a much greater level of complexity across bacterial genera. Thus, whereas the MscL channel activity is usually the product of a single, moderately conserved, gene, many organisms possess multiple MscS homologues. Possession of multiple channels that are differentially expressed may offer the cell a graded response to hypoosmotic shock.

Over the course of evolution different organisms have fashioned unique solutions that reflect their environmental niche. Some organisms that spend all of their ‘normal’ life in marine environments have lost MscL [36,37^•^] (LE Lehtovirta and GW Nicol, personal communication). Some marine organisms can be rescued from severe hypoosmotic stress by transgenic expression of MscL from *E. coli*. An important pair of questions relating to this observation are — why do *Vibrio alginolyticus* and *Salinispora tropica* not express their MscS channels (they both lack MscL) sufficiently to protect themselves against hypoosmotic shock [37^•^,38] and what roles do their MscS-type channels play? The variation in *Vibrio* species has previously been commented upon [2^•^] and recent work has identified that *Campylobacter jejuni*, uniquely among all the members of this species, has no MscL [26^•^]. Intriguingly, the last example has provided the first demonstration of a role for MscS in pathogenicity [26^•^]. Bacteroid formation between symbiotic bacteria and plants may afford further examples of other critical roles for MscS homologues. Differential expression of homologues during bacteroid development may be indicative of a need for protection during morphological changes [39]. Note, however, that is not a general rule — in other symbioses expression of MscS is repressed during formation of bacteroids [40]. As in all biological systems, evolutionary history is neither obvious nor simple but has determined the current observable properties of cells. One can state the generality with confidence but the individual solutions to problems often involve greater subtlety.

Added to the complexity of diversity are the problems of differential expression of MS channels and the signals that control the transcription of their structural genes. A limited amount has been learned about the *E. coli* MS channels [28^••^,41], but more insight is needed to complete the understanding of the physiology of MS channels. Recent work has highlighted the unexpected abundance of MscL channels, 300–1000 per cell, in *E. coli* [27^••^]. Newer methodologies have the potential to revise these numbers downwards (GW Li, personal communication), but there would still be an apparent over-sufficiency of channel capacity. Calculations have indicated that a single open MscL channel could suffice to deplete the cell of its ion pools in 1 s [42] (and thus that 5–10 channels opening would meet this requirement in 100–200 ms, the time line for adaptation; Figure 2). This calculation, however, allowed the membrane potential to remain constant (−100 to −180 mV) during gating. Opening any MS channel for a brief period would depolarise the membrane completely and thus the flow through the channel may have been over-estimated at least 10-fold (see Box 1). The other channels have much lower maximum conductance [28^••^] and thus can carry much lower ion flows. Thus, one explanation for the synthesis of large numbers of channels may be that they meet the requirement for *rapid* voiding of the cytoplasmic solutes, on the timescale <150 ms, in response to hypoosmotic stress (see Box 1; Figure 2). Multiple channel types provide a temporal response for less severe hypoosmotic shock and the abundance allows rapid reduction of the turgor pressure.

## Concluding remarks

Given the prominent role of MS channels in preserving bacterial cell wall integrity it would not be unexpected if integration of MS channel gating with cell wall biosynthesis were to emerge as a theme. No evidence for such a connection exists. The last few years have seen a step-change in our understanding of the integration of the outer membrane with PTG biosynthesis through specific connections made between lipoproteins and biosynthetic complexes that stimulate synthesis of new wall material [43^••^]. The molecular dimensions of the two sets of proteins, one anchored in the cytoplasmic membrane and the other in the outer membrane, provide the cell with a measure of the dimensions of the periplasm. This mechanism may exist to ensure synthesis at the appropriate positions in the cell.

Does this connect in any way with MS channels? The immediate answer is that no explicit data suggest a connection. Thus, the *E. coli* mutant lacking all seven MS channels has no obvious growth or division defect (SS Black *et al.*, unpublished data). Moreover, similar channel-free mutants have been made in other organisms with no reported growth defects [24,25,26^•^]. One aspect of channel structural diversity may ultimately find an explanation through integration of these core functions of cells. Thus, MS homologues differ in the number of transmembrane helices, in the presence or absence of a periplasmic domain and in modifications to the carboxyterminal domain [31,44,45^•^]. Among these variations the periplasmic domains are intriguing because they have unique sequences, found only in closely related species and genera, indicating potential co-evolution with other cell wall components [13^•^]. Structure prediction is hazardous and no consistent patterns have emerged at this time. However, a preliminary analysis of the MscS homologue in *Rhodopseudomonas palustris* suggests a structural similarity to PTG synthetic complexes (IR Booth, unpublished data). At this time it is an interesting correlation and must be consolidated through further analysis.

In conclusion water flow across the membrane is essential to life, but provides a challenge to the physical integrity of the bacterial cell. MS channels have evolved to meet that challenge. In higher organisms that role has evolved into a specific function in preserving the integrity of chloroplasts [1^•^]. Thus, the proposed prokaryotic origin of eukaryotic organelles finds a further manifestation in MS channels!

## References and recommended reading

Papers of particular interest, published within the period of review, have been highlighted as:

• of special interest
•• of outstanding interest

## Box 1 Ion flows through MS channels for osmotic adjustment.

Before channel gating, *E. coli* cells adapted to 0.5 m NaCl in defined medium may contain ∼500 mm osmotically active K^+^ (see text, some K^+^ will be bound by fixed anions), ∼250 mm glutamate and ∼200 mm trehalose plus ∼200 mm metabolic anions (e.g. intermediates in glycolysis and the TCA cycle) (precise values vary slightly from strain to strain) [6,35]. This corresponds to ∼7 × 10^8^ osmotically active solutes/cell based on Avogadro's number and a volume of 10^−15^ L/cell. After shock, cells would be expected to contain ∼2–3 × 10^8^ osmotically active solutes. Thus, to become balanced with a medium of ∼200 mOsm the cells must lose ∼5 × 10^8^ osmotically active solutes in a few ms. On the basis of previous calculation [42], and on the conductance of MscS (∼1 nS at 20 mV potential difference) one can estimate ion movement through a single MS channel of this type as ∼3 × 10^5^ ions (solutes)/ms. [Note that channel gating would lead to rapid inflow of Na^+^ and H^+^ down their gradients, as well as the outward movement of solutes [35], collapsing the membrane potential. Thus flux through any individual channel may be substantially slower.] Consequently, total adaptation to osmolarity could, theoretically, be achieved by 1000 such channels in 1 ms or by 50 channels in 20 ms (this number can be adjusted up or down for lower or higher channel conductance, such as YnaI [28^••^] or MscL [22], respectively). Indeed these data come close to explaining the apparent superabundance of MscL channels [27^••^] — namely this level of expression allows very rapid voiding of solutes (Figure 2), consistent with preventing breaches of the cell wall. It has been shown that opening MS channels causes rapid equilibration of internal and external solutes [35], leading to replacement of K^+^ with Na^+^ and H^+^. Consequently bacteria must be very careful when gating their channels at low pH [4].
